# Low doses of caffeine reduce heart rate during submaximal cycle ergometry

**DOI:** 10.1186/1550-2783-4-11

**Published:** 2007-10-09

**Authors:** Steven R McClaran, Thomas J Wetter

**Affiliations:** 1 Department of Kinesiology& Health, University of Wisconsin Oshkosh, Oshkosh, WI 54901, USA; 2 School of Health Promotion & Human Development, University of Wisconsin-Stevens Point, Stevens Point, WI 54481, USA

## Abstract

**Background:**

The purpose of this study was to examine the cardiovascular effects of two low-levels of caffeine ingestion in non habitual caffeine users at various submaximal and maximal exercise intensities.

**Methods:**

Nine male subjects (19–25 yr; 83.3 ± 3.1 kg; 184 ± 2 cm), underwent three testing sessions administered in a randomized and double-blind fashion. During each session, subjects were provided 4 oz of water and a gelatin capsule containing a placebo, 1.5 mg/kg caffeine, or 3.0 mg/kg caffeine. After thirty minutes of rest, a warm-up (30 Watts for 2 min) the pedal rate of 60 rpm was maintained at a steady-state output of 60 watts for five minutes; increased to 120 watts for five minutes and to 180 watts for five minutes. After a 2 min rest the workload was 180 watts for one minute and increased by 30 watts every minute until exhaustion. Heart rate (HR) was measured during the last 15-seconds of each minute of submaximal exercise. Systolic blood pressure (BP) was measured at rest and during each of the three sub-maximal steady state power outputs. Minute ventilation (V_E_), Tidal volume (V_T_), Breathing frequency (Bf), Rating of perceived exertion (RPE), Respiratory exchange ratio (RER), and Oxygen consumption (VO_2_) were measured at rest and during each minute of exercise.

**Results:**

Caffeine at 1.5 and 3.0 mg/kg body weight significantly lowered (p < 0.05) HR during all three submaximal exercise intensities compared to placebo (range – 4 to 7 bpm lower) but not at rest or maximal exercise. BP was significantly higher (p < 0.05) at rest and after the 3 mg/kg caffeine vs placebo (116 ± 13 vs 123 ± 10 mm Hg). Neither dose of caffeine had any effect on BP during submaximal exercise. Caffeine had no effect on V_E_, V_T_, VO_2_, RPE, maximal power output or time to exhaustion.

**Conclusion:**

In non habitual caffeine users it appears that consuming a caffeine pill (1.5 & 3.0 mg/kg) at a dose comparable to 1–3 cups of coffee lowers heart rate during submaximal exercise but not at near maximal and maximal exercise. In addition, this caffeine dose also only appears to affect systolic blood pressure at rest but not during cycling exercise.

## Introduction

Caffeine is the most widely used drug in the world and the use of caffeine or caffeine containing beverages is extremely common among all levels of athletes hoping to gain an ergogenic benefit. Previous investigations have shown that caffeine enhances short-term power, short-term high-intensity time to exhaustion, resistance to fatigue, as well as endurance performance [1,2]. Thus caffeine is generally regarded to have ergogenic properties and its use is currently banned by the NCAA at dose which elicits a urinary concentration of 15 ug/ml; a dose which is far higher than needed to achieve performance benefits [2]. Much of the exercise research with caffeine has investigated potential mechanisms mediating performance. Enhanced fat oxidation leading to glycogen sparing has historically been a popular theory for the improvement in endurance performance however this mechanism might be incomplete. The inhibition of adenosine receptors including those in the central nervous system is now considered by some to be a more likely mediator of caffeine's ergogenic properties [1-3]. Some have added to the debate suggesting that the inhibition of adenosine receptors including those in the central nervous system could be a more likely mediator of caffeine's ergogenic properties [1-3]. While not considered to have a role in performance enhancement, caffeine's cardiovascular effects have also generated much interest. Much of this research however, has been conducted in resting subjects and has investigated the potential negative health consequences from caffeine-induced elevations in blood pressure [4].

Investigations reporting the effect of caffeine on heart rate during exercise are equivocal, with some indicating increases [5-9], decreases [10-12], and no effect [13-28]; while those reporting blood pressure have typically found higher values [9,11,16,17,20,22,25]. Caffeine dose, exercise intensity, and caffeine habituation are all factors which differ among these studies. Despite the fact that the data is inconclusive, statements still reflect the common notion that caffeine increases heart rate during exercise and this type of statement is often made without reference to dose, intensity of activity, or habituation status.

Because the ergogenic effect of caffeine has been shown to occur at a dose lower than what has been typically used in research studies [2], many athletes are likely using caffeine at more moderate doses (< 5 mg/kg). Therefore investigations of the various cardiovascular effects of caffeine at these lower doses are important. In addition, during their training bouts and in competition many athletes use heart rate monitors as an indicator of their optimal pace, therefore the effect of caffeine on heart rate could be of great importance to the athlete. Thus, the purpose of this study was to measure the cardiovascular effects of two low-levels of caffeine ingestion (in pill form) in non habitual caffeine users at various submaximal and maximal exercise intensities.

## Methods

### Subjects

Nine male college subjects, age 19–25 yr, body weight (83.3 ± 9.3 kg) and height (184 ± 6 cm), volunteered to participate in the study. All subjects were normotensive, indicated non-habitual caffeine use (via a questionnaire), and were aerobically active at the time of the study (VO_2max_: 49.7 ± 6.3 ml/kg/min). All subjects were healthy at the time of the study without any history of cardiovascular or lung disease and none of the subjects was a smoker. The protocol was explained along with possible risks and discomforts and informed consent was obtained in writing from each subject before testing. All procedures were approved by the Institutional Review Board for the Protection of Human Subjects of the University of Wisconsin-Stevens Point.

### Protocol

Using a double-blind protocol each subject completed three experimental trials in random order. Trials were conducted at the same time of day and were separated by at least 48 hours. Subjects were asked to refrain from heavy exercise the day before each trial and to keep the time of their last meal consistent between each trial. Upon reporting to the laboratory for each trial, subjects closed their eyes and were given approximately 4 oz of water and a gelatin capsule containing either a placebo, 1.5 mg/kg caffeine (CAF1.5 – equivalent to about 1.2 cups of coffee [2]), or 3.0 mg/kg caffeine (CAF3). The subjects then rested quietly for the next 30 minutes. During this time, subjects filled out a survey which listed caffeine-containing products, and indicated amounts (if any) of consumption of these products in the previous 48 hours. Six subjects indicated no caffeine product use and three others reported use of ≤ 106 mg in the last 48 hours.

Each subject completed their three exercise trials on a Monark (Model 828 E) bicycle ergometer. For each exercise session, after a warm-up period (30 Watts for 2 min) a pedal rate of 60 rpm was maintained for the remainder of the test. For the first phase the workload was initially set at a steady-state power output of 60 watts (~30% of VO_2max_) for five minutes; the workload was then increased to 120 watts (~46 of VO_2max_) for five minutes and then to 180 watts (~64% of VO_2max_) for five minutes. The second phase of the exercise protocol started after a 2 min rest. The subjects were quickly brought back up to 180 watts for one minute again maintaining a pedal rate of 60 rpm. The workload was then increased by 30 watts every minute until exhaustion (with exhaustion being determined when the 60 rpm pedal rate was no longer able to be maintained). The subjects then were then cooled down to ensure safety of the trial.

### Measurements

Heart rate (HR) was measured using a Polar heart rate monitor (Polar Beat, Stamford, CT). Heart rates were recorded during the last 15-seconds of each minute of exercise with both the fourth and fifth minute measurements during submaximal exercise used for comparisons. Minute ventilation (V_E_), Tidal volume (V_T_), breathing frequency (Bf), respiratory exchange ratio (RER), and oxygen consumption (VO_2_) was measured using a Sensor Medics Metabolic Cart (model Vmax Spectra) at rest and during each minute of exercise. Systolic blood pressure (BP) was measured by auscultation over the left brachial artery once at rest and twice during each of the three sub-maximal steady state power outputs with both measurements used for comparison; BP was not measured during the second phase including maximal exercise. Rating of perceived exertion (RPE) using a Borg category scale (6–20) was recorded in the last 30-seconds during each of the three sub-maximal steady state power outputs and at maximal exercise. The final workload achieved and the time at that workload was recorded as an indication of potential performance differences.

### Statistics

All data are reported as mean ± SD. Data were analyzed using a 2 × 2 (caffeine/placebo x rest/exercise) analysis of variance ANOVA with repeated measures in which the subjects served as their own control. Mauchly's Test of Sphericity was used to determine if there was a violation. To correct for any violation of the sphericity assumption with the repeated factors, a Greenhouse-Geisser correction was applied to the F-ratio. When the ANOVA yielded a significant F-ratio, a post hoc pairwise comparison of the factor was completed. All analyses were performed with SPSS for Windows version 13.0 statistical package (SPSS Inc, Chicago, IL). Statistical significance for all tests was set at p ≤ 0.05.

## Results

The data for the metabolic variables after 30 minutes of rest can be seen in Table 1. RER was significantly lower for the 3 mg/kg caffeine dose vs the placebo trial (p < 0.05). There were no differences in the resting mean V_E_, VO_2_, V_T_, and Bf between the three trials.

**Table 1:** Respiratory and Metabolic values at rest

	Placebo	1.5 CAF	3.0 CAF
V_E _(L·min^-1^)	13.4 ± 3.1	13.3 ± 2.3	14.2 ± 3.0
Bf (breaths·min^-1^)	16.2 ± 4.6	14.6 ± 2.4	16.6 ± 3.9
V_T _(L·breath^-1^)	0.93 ± 0.26	0.99 ± 0.20	0.95 ± 0.18
VO_2_(ml·kg^-1^·min^-1^)	5.2 ± 0.8	5.6 ± 1.0	5.7 ± 0.8
RER	0.89 ± 0.08	0.86 ± 0.09	0.84 ± 0.09*

Values listed as mean ± SD. V_E_, ventilation; Bf, breathing frequency; V_T_, tidal volume; VO_2_, oxygen consumption; RER, respiratory exchange ratio. PLA, placebo; 1.5 and 3.0 represent dose of caffeine in mg·kg body weight^-1^. * Significantly different from placebo (p < 0.05).

**Figure 1:**
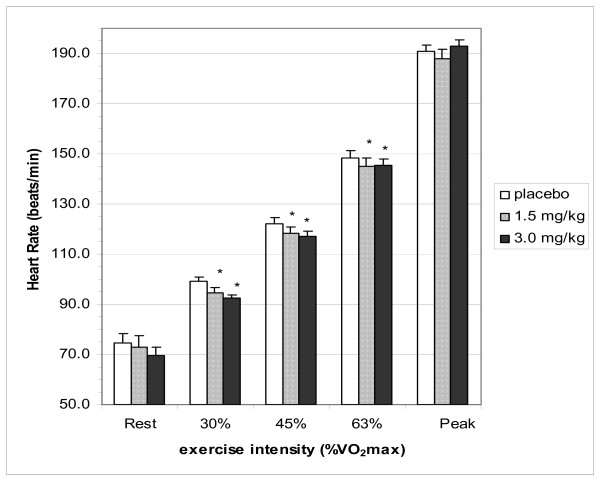
**Heart rate at rest and during exercise**. Differences in heart rate between placebo and caffeine trials. Placebo, 1.5 and 3.0 represent dose of caffeine in mg·kg body weight^-1^. Values are listed as mean ± SE. * = the mean difference compared to placebo is significant (p < 0.05).

The HR responses for rest, the three submaximal intensities and at maximal exercise can be seen in Figure 1. HR tended to be lower at rest after administration of CAF3 vs placebo, however this was not statistically significant (p = 0.08). However the HR for both the CAF1.5 and the CAF3 trials were significantly lower (p < 0.05) vs the placebo trial for all three submaximal intensities (the range was 4 to 7 bpm lower). This lower heart rate effect was not present during the higher intensities used for the incremental test (data not shown) or at maximal exercise. There were no differences between the CAF1.5 vs the CAF3 trials during submaximal or maximal exercise.

**Figure 2:**
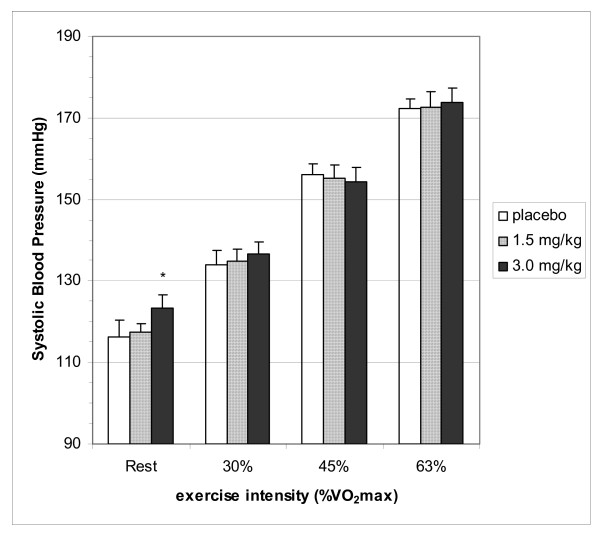
**Systolic blood pressure at rest and during exercise**. Differences in Systolic blood pressure between placebo and caffeine trials. Placebo, 1.5 and 3.0 represent dose of caffeine in mg·kg body weight^-1^. Values are listed as mean ± SE. * = the mean difference compared to placebo is significant (p < 0.05)

The mean BP responses for rest and the three submaximal intensities are displayed in Figure 2. Resting systolic BP was significantly higher (p < 0.05) after the CAF3 dose vs placebo trial (116 ± 13 mmHg vs 123 ± 10 mmHg). Neither dose of caffeine had any effect on BP during submaximal exercise.

The mean respiratory and metabolic values during submaximal and maximal exercise are displayed in Table 2. RER was not different between trials for the lowest exercise intensity (30% of VO_2max_). RER with CAF3 added was significantly lower (p < 0.05) compared to both the CAF1.5 and placebo trials at 45% of VO_2max_. RER with CAF3 was significantly lower (p < 0.05) compared to the CAF1.5 trial at 63% of VO_2max_. During maximal exercise RER with CAF3 was significantly lower (p < 0.05) compared to the placebo trial. There were no significant differences for V_E_, VO_2_, V_T _or RPE during the submaximal workloads or at maximal exercise between trials. Bf was significantly lower (p < 0.05) at 45% of VO_2max _with CAF3 vs placebo. However there were no significant differences for Bf during any other submaximal workloads or at maximal exercise. There were also no differences between the trials in the peak workload or in time to exhaustion at their maximum workload.

**Table 2:** Respiratory and metabolic values during submaximal and maximal exercise

**% of VO**_2max_	30	45	63	Peak VO_2_
V_E _(L·min^-1^)				
PLA	28.0 ± 2.3	41.5 ± 2.4	56.6 ± 3.4	129.0 ± 15.8
1.5	28.6 ± 3.0	42.7 ± 3.8	59.3 ± 5.3	129.1 ± 13.4
3.0	28.5 ± 2.4	42.0 ± 3.2	59.5 ± 6.0	127.1 ± 12.3
Bf (breaths·min^-1^)				
PLA	19.3 ± 2.8	21.3 ± 2.8	23.5 ± 4.1	41.0 ± 8.3
1.5	18.7 ± 3.1	20.8 ± 3.2	23.2 ± 4.4	38.8 ± 6.0
3.0	18.0 ± 2.6	20.0 ± 3.3*	23.3 ± 4.1	38.9 ± 6.7
V_T _(L·breath^-1^)				
PLA	1.55 ± 0.23	2.02 ± 0.27	2.50 ± 0.41	3.21 ± 0.36
1.5	1.63 ± 0.27	2.13 ± 0.28	2.64 ± 0.36	3.38 ± 0.31
3.0	1.67 ± 0.19	2.22 ± 0.42	2.63 ± 0.41	3.32 ± 0.36
VO_2_(ml·kg^-1^·min^-1^)				
PLA	14.9 ± 1.6	22.6 ± 2.4	31.0 ± 3.3	49.7 ± 4.4
1.5	14.8 ± 0.9	22.7 ± 1.7	31.4 ± 2.7	50.7 ± 5.6
3.0	15.2 ± 1.0	23.0 ± 2.1	31.9 ± 3.2	50.3 ± 4.1
RER				
PLA	0.85 ± 0.04	0.90 ± 0.02	0.94 ± 0.03	1.21 ± 0.05
1.5	0.86 ± 0.05	0.92 ± 0.03	0.96 ± 0.04	1.18 ± 0.06
3.0	0.82 ± 0.06	0.88 ± 0.05^*†^	0.93 ± 0.03^†^	1.16 ± 0.06^*†^
RPE				
PLA	8.4 ± 1.3	11.7 ± 1.5	13.8 ± 0.9	19.0 ± 0.6
1.5	8.2 ± 1.3	11.0 ± 0.7	13.4 ± 1.0	19.1 ± 0.6
3.0	7.9 ± 1.1	11.3 ± 1.2	13.4 ± 1.0	19.3 ± 0.6

Values listed as mean ± SD. V_E_, ventilation; Bf, breathing frequency; V_T_, tidal volume; VO_2_, oxygen consumption; RER, respiratory exchange ratio; RPE, rating of perceived exertion. PLA, placebo; 1.5 and 3.0 represent dose of caffeine in mg·kg body weight^-1^.

* Significantly different from placebo (p < 0.05).

^† ^Significantly different from 1.5 caffeine in mg·kg body weight^-1^. (p < 0.05).

## Discussion

The results of this study provide evidence that caffeine at low doses of 1.5 and 3.0 mg/kg body weight significantly decreased heart rate during low to moderate intensity cycle exercise in non-habitual caffeine users. These low doses of caffeine did not significantly affect heart rate at rest nor did they affect performance, HR, and BP at maximal exercise. Our results differ from other studies in that systolic blood pressure was not higher in our subjects with caffeine compared to control during exercise. At these doses, caffeine had no effect on V_E_, V_T_, VO_2_, RPE, maximal power output and time to exhaustion. Therefore those young adult athletes that use heart rate monitors as an indicator of their optimal pace should be aware of the effect of caffeine lowering effect on heart rate during submaximal exercise.

Investigating the cardiovascular effects of caffeine is not new. In 1930, Grollman [29] had individual subjects ingest various doses of caffeine and measured heart rate, blood pressure, VO_2 _and cardiac output. Because caffeine has been found to cause an increase in blood pressure, the potential negative health consequences of caffeine consumption are often a main focus of these studies. The majority of studies that have investigated cardiovascular effects of caffeine during exercise have used higher doses (≥ 5 mg/kg body weight) and whether the studies utilized habituated or non-habituated subjects has varied. To examine the potential role that study design differences (specifically dose, habituation status, and exercise intensity) might contribute to the equivocal cardiovascular effects of caffeine during exercise, we reviewed the literature for studies which reported the effects of caffeine on heart rate and systolic blood pressure during exercise (Table 3).

**Table 3:** Summary of previous studies of caffeine effects during submaximal exercise

	**Study**	**Caffeine use**	**Caffeine**	**% of VO2max**	**Mode**	**Effects of caffeine on HR**	**Systolic Blood Pressure**
A	Bangsbo (1992)	n = 11 not reported	500 mg	80–100	Treadmill	No diff	Not studied
B	Bell (1998)	n = 8 habitual	5 mg/kg	85	Cycle	**↑ **at 5 min; not at 10 min	Not studied
C	Bell (2002)	n = 8 non-habitual n = 13 habitual	5 mg/kg	50 & 80	Cycle	No diff at 50% **↑ **at 80% for both h + nh	Not studied
D	Bell et al (2003)	n = 9 habitual	5 mg/kg	80	Cycle	No diff	Not studied
E	Casal et al (1985)	n = 9 not reported	6 mg/kg	75	Treadmill	No diff	Not studied
F	Daniels et al (1998)	n = 10 non-habitual	6 mg/kg	65	Cycle	No diff	**Higher with caffeine**
G	Engels et al (1999)	n = 8 habitual	5 mg/kg	30	Cycle	No diff	**Higher with caffeine**
H	Flinn et al (1990)	n = 9 non-habitual	10 mg/kg	40–100	Cycle	No diff	Not studied
I	French et al (1991)	n = 6 non-habitual	10 mg/kg	75	Treadmill	No diff	Not studied
J	Gaesser GA (1985)	n = 8 habitual	5 mg/kg	37–100	Cycle	**↓ at 37–77% max, **no diff at max	Not studied
K	Graham et al (2000)	n = 9 non-habitual	6 mg/kg	70	Cycle	No diff	**Higher with caffeine**
L	Jacobsen et al (2001)	n = 8 non-habitual	6 mg/kg	70	Cycle	No diff	Not studied
M	Kaminsky (1998)	n = 8 non-habitual n = 8 habitual	5 kg/mg	30, 50, 70	Treadmill	No diff	**Higher for both groups with caffeine**
N	McNaughton (1987)	n = 10 non-habitual	10 + 15 mg/kg	Not reported	Cycle	****↑ **Higher with caffeine**	Not studied
O	Nishijima (2002)	n = 8 habitual	300 mg	40–50	Cycle	Not measured	No diff
P	Perkins R (1975)	n = 14 not reported	4 + 7 + 10 mg/kg	50–100	Cycle	No diff for any doses	Not studied
Q	Powers (1983)	n = 7 not reported	5 mg/kg	25 – 100	Cycle	No diff	Not studied
R	Sasaki (1987)	n = 7 not reported	3.2 mg/kg	63	Treadmill	**↑ Higher with caffeine**	Not studied
S	Sullivan et al (1992)	n = 5 non-habitual n = 5 habitual	3.3 mg/kg	45	Treadmill	**↓ for non-habitual** No diff for habitual	**Higher for both groups with caffeine**
T	Sung et al (1990)	n = 34 habitual	5 mg/kg	25 & 50	Cycle	No diff	**Higher with caffeine**
U	Sung et al (1995)	n = 12 habitual	3.3 mg/kg	Not reported	Cycle	No diff	**Higher with caffeine**
V	Tarnopolosky (1989)	n = 6 habitual	6 mg/kg	70	Treadmill	No diff	Not studied
W	Trice et al (1993)	n = 8 habitual	5 mg/kg	85–90	Cycle	No diff	Not studied
X	Turley & Gerst (2006)	n = 52 not reported	5 mg/kg	65% of max hr 75% of max hr	Cycle	**↓ **for boys & girls at 65% **↓ **for boys & girls at 75%	**↑ **for boys at 65% **↓ **for girls at 75%
Y	Weir et al (1987)	n = 6 not reported	6.5 mg/kg	72–76	Treadmill	No diff	Not studied
McClaran (present study)		n = 9 non-habitual	1.5 + 3 mg/kg	30, 46, 63	Cycle	**↓** at 30-63% max	No diff

All studies used adults as subjects except Turley & Gerst (2006)

Comparing the results of the current study to previous studies is somewhat difficult given the different doses of caffeine, types and intensities of exercise, and the varied use of either habituated or non-habituated subjects. However, our finding of a lower heart rate with caffeine use during exercise is in agreement with three previous studies. Sullivan et al. [11] studied five habituated and five non-habituated subjects who walked at 46% of VO2max and observed that the five non-habituated subjects showed a significantly lower heart rate after administration of 3.3 mg/kg caffeine (133.9 vs 143.7 during placebo) whereas the difference was not significant in the five habituated subjects (121.8 vs 124.4). Gaesser and Rich [10] gave 5 mg/kg caffeine to low to moderate caffeine users and found lower heart rates (6–10 bpm) compared to placebo at cycling exercise intensities between 30–70% of VO_2max_. They found no difference in HR above 75% of VO_2max _(HR > 160 bpm). More recently, Turley and Gerst [12] found lower heart rates in young boys and girls during cycle exercise at intensities of 41% and 64% of VO_2max _(boys) and 51% and 77% of VO_2max _(girls) after a caffeine dose of 5 mg/kg. Of note is that Sullivan et al. [11] used 3.3 mg/kg caffeine and the other two [10,12] used a higher dose of caffeine (5 mg/kg). Further investigation would seem warranted with different levels of caffeine and comparing non-habituated vs. habitual caffeine users.

Our results are in contrast to a number of studies that have reported no effect of caffeine administration on exercise heart rate [13-28]. A possible explanation is that with one exception [23] each of the remaining 15 studies used a ≥ 5 mg/kg dose of caffeine. Of note is that Perkins used doses of 4, 7, and 10 mg/kg and found no heart rate difference between any of the trials compared to control for exercise intensities at 50% of VO_2max _through maximal exercise. The development of tolerance to the cardiovascular effects of caffeine in habitual users could also be a potential confounder [1,2]. For example Robertson [30] reported that three days of caffeine administration abolished the pressor effect of caffeine in previously caffeine naive subjects. From our review of the caffeine studies in Table 3 the role of habituation is unclear: In the studies we cited finding no change in heart rate during submaximal exercise, five used habituated subjects [14,16,25-27] and five used non-habituated subjects [16,18-21]. The remaining studies did not report the habituation of their subjects [13-15,23,24,28]. Furthermore Kaminsky [22] found no change in heart rate during submaximal exercise between their eight habituated vs eight non-habituated subjects with administration of 5 mg/kg of caffeine.

Our results are also in direct contrast to several studies which have found higher heart rates with caffeine during exercise. McNaughton [7] observed higher heart rates during cycling at workloads from 50 to 300 watts after high doses (10 and 15 mg/kg) of caffeine compared with placebo. Sasaki [8] also found a significant increase in HR with caffeine vs placebo but only during the last 30 minutes of a 2 hr running bout (intensity – 62–67% VO_2max_). With a similar low dose of caffeine used in our study (3.3 mg/kg), Sung [9] found a higher heart rate response to a 67 watts workload in hypertensive but not normotensive men. Finally, Bell [5,6] found that a dose of 5 mg/kg (in both habitual and non-habitual users) resulted in a significant increase in HR at 80% and 85% VO_2max _but no effect of caffeine on HR during cycling at a lower intensity (50% of VO_2max_). Of note is that a separate previously mentioned study by Bell [14] found no difference in heart rates at 80% of VO_2max_. Considering the possible factors that might account for these findings not in agreement with the present study, the intensity of exercise and the higher dose of caffeine used may be the most likely. However differences in mode of exercise, duration, habituation to caffeine, hypertension status, and perceived stress could also be confounders.

Even though cardiac output was not measured in this study, the similar levels in VO_2 _during each of the trials would lead us to speculate that there was a comparable cardiac output with the placebo and caffeine trials. Pincomb [31] at rest and both Engels [17] and Sullivan [11] during exercise measured cardiac output and found no difference on the caffeine compared to the placebo days. Therefore we would further speculate that the lower heart rate we observed during moderate exercise would indicate an increased or optimized stroke volume. The most likely mechanism would seem to be either an enhanced contractility or a higher preload with caffeine use during submaximal exercise in non-habituated subjects. Gould et al. [32] showed an augmented (but not significant) stroke volume with caffeine ingestion due to an enhanced left ventricular end-diastolic volume. Robertson [33] speculates that caffeine's effect may be due to an enhancement of myocardial contractility caused by methylxanthines.

Another possible explanation of a lowered HR is due to the baroreflex reflexively lowering heart rate in response to an elevation of BP in an attempt to reestablish "normal" BP [34]. Perhaps caffeine habituation might make this reflex less sensitive and therefore result in no HR change (and an increase in BP). In support of this, Sullivan [11] found HR decreases in non-habitual users but not in habitual users, whereas the current study used non-habitual users. While Turley and Gerst [12] did not report caffeine use, because their subjects were children we might assume that caffeine use was highly variable. However, Gaesser and Rich [10] used subjects who were low to moderate caffeine users (100–200 mg/kg daily who abstained from caffeine in the previous 24 hours) and found a lower heart rate during exercise with caffeine. Moreover, there has been previous mention of the studies that used non-habituated caffeine users and showed similar or higher heart rates after caffeine administration.

A final influence on the effect of caffeine on HR is the effect that caffeine may have in the face of "stress". Coffee has been shown to increase the HR response to mental stress [35]. The tendency of caffeine alone to reduce heart rate appears to be reversed when caffeine is consumed during active coping stress. Rest and low to moderate intensity exercise may be perceived as not very stressful, especially in healthy and fit people. Our data indicated that the effect of CAF on HR was not evident during higher intensity exercise, perhaps because of the perception of higher stress.

Lastly it is unclear as to why the lower heart rate during submaximal exercise with the 1.5 and 3 mg/kg caffeine trials disappears during near maximal and maximal exercise. This similar heart rate response at high levels of exercise is consistent with other studies using either habituated or non-habituated subjects [10,13,14,18,23,24,27]. Based on the previous discussion it could be that contractility or preload cannot be further optimized at high exercise intensity and therefore the caffeine effect is negated. Further follow-up caffeine studies measuring stroke volume would need to be conducted in order to fully illuminate this question.

We observed that systolic blood pressure was significantly higher at rest which is in agreement with most previous studies [4,11,16,17,20,22,25]. In a review Myers [4] notes that most of the recent studies at that time showed an increase in blood pressure with caffeine at rest. He suggested that the mechanisms of the higher blood pressure response to caffeine could mostly be attributed to a pressor effect caused by a sympathetic mediated vasoconstriction. It has also been postulated that the higher systolic blood pressure occurs because caffeine blocks the normal vasodilatory effects of adenosine which can also lead to peripheral vasoconstriction [11,17]. There remains more to be done before caffeine's mechanism of action on systolic BP is completely understood.

At the doses given in the current study caffeine had no effect on systolic BP during exercise and this result differs from previous studies showing increased systolic BP during submaximal exercise intensity [1,11,16,17,22,25,36]. As discussed previously when comparing the heart rate response, the difference in our findings could possibly be explained by the mitigating effect of habitual caffeine use. However it should be noted that the previous studies using non-habituated subjects [11,16,22] show a significantly higher systolic BP. Furthermore Kaminsky [22] and Sullivan [11] who compared habituated to non-habituated subjects saw the same higher systolic BP response to caffeine in both groups. The above studies used different doses (between 3.3 mg/kg – similar to the present study and 6 mg/kg) and consistently found a higher systolic BP during submaximal exercise; therefore the amount of caffeine administered does not seem a likely explanation. A closer analysis of our individual data revealed the following: Of the nine subjects tested, four showed a consistently higher systolic BP response with 3 mg/kg caffeine, three were consistently lower and the last two had equivocal systolic BP data. Therefore there does not seem to be an easy explanation as to why there is a discrepancy between ours compared to other studies.

The timing of caffeine administration before the beginning of exercise is yet another factor which may influence cardiovascular effects. According to Graham [1] most investigators have their participants take the caffeine dose 60 minutes prior to exercise. Looking at the studies in Table 3 the range is anywhere from immediately before exercise up to waiting three hours before exercise, with the most common lag time from 40–60 minutes. Robertson [33] showed however that the effect of caffeine (250 mg) on resting heart rate was biphasic. An initial decrease occurred between 45 and 75 minutes after administration, followed by a mild increase above baseline levels beginning at 105 minutes. Whether this effect would be altered by caffeine dose or would persist during exercise is unknown. However, the heart rates taken during our submaximal exercise bouts occurred within the window where caffeine elicited a drop in heart rate.

## Conclusion

A review of lay press and scientific literature indicates a frequently made statement that caffeine ingestion increases heart rate. In an article for public education on the MayoClinic.com website [37] entitled "Caffeine: Does it help you lose weight?" a sentence reads: "Also, keep in mind that caffeine is a stimulant that can increase your heart rate ..." While technically accurate (contains the word "can"), it does not indicate at what dose this might likely be true. The association of caffeine and increased HR is also often reported in the scientific exercise and sports medicine literature. In a recent review article: "Caffeine as an Ergogenic Aid", Keisler and Armsey [3] state "In the cardiovascular system, caffeine acts to increase heart rate and blood pressure." Again, at what dose, and whether the authors are speaking about resting or exercise heart rate, is unclear from the text. In the discussion section of their research study, Jacobson et al. [21] state, "There is general agreement that caffeine ingestion increases exercise heart rate because of its powerful effect on the sympathetic nervous system." For this statement the authors cite two references however, in neither cited study were heart rates reported. These accounts are clearly contradictory to the majority of the research literature and perpetuation of the notion that caffeine increases heart rate during exercise seems unwarranted. In addition we noticed that most of the recent studies that compared heart rates during exercise with caffeine administration have typically cited only a few studies and have not considered the breadth of the possible issues. While not a complete review of all the relevant studies we feel that the studies we presented in Table 3 suggest there is more that needs to be done before there is general agreement on the caffeine effect on heart rate and blood pressure during exercise.

Further investigations of the cardiovascular effects of caffeine are especially warranted with the recognition that its performance enhancing effects on endurance exercise can occur at doses near or below 3 mg/kg [1,38,39]. Already the average American consumes 238 mg of caffeine per day [1], however even more athletes may use these low caffeine doses to obtain the ergogenic benefit. Our study suggests in young, healthy subjects that a caffeine pill comparable to 1–3 cups of coffee lowers heart rate during submaximal exercise and therefore may alter workload selection when using a heart rate monitor as an index for exercise intensity. Clearly, clarification of caffeine's cardiovascular effects at these low but effective doses is important. In addition, the cardiovascular effects of caffeine with different exercise intensities, mode, sex, age and fitness as well as the mechanism(s) responsible for cardiovascular effects remains to be clarified. In addition further research could be completed that specifically determines if there are differences between habituated vs. non-habituated users and also that compares the effect of various other caffeine doses (e.g., 4, 5 & 6 mg/kg caffeine).
